# Involvement of Kv1.5 Protein in Oxidative Vascular Endothelial Cell Injury

**DOI:** 10.1371/journal.pone.0049758

**Published:** 2012-11-21

**Authors:** Wen-Liang Chen, Xiong-Qing Huang, Li-Yan Zhao, Jie Li, Jian-Wen Chen, Ying Xiao, Yun-Ying Huang, Jie Liu, Guan-Lei Wang, Yong-Yuan Guan

**Affiliations:** 1 Department of Pharmacology, Guangzhou Medical University, Guangzhou, P. R. China; 2 Department of Anesthesiology, The First Affiliated Hospital, Sun Yat-sen University, Guangzhou, P. R. China; 3 Department of Pharmacology, Zhongshan School of Medicine, Sun Yat-sen University, Guangzhou, P. R. China; 4 Department of Anesthesiology, Sun Yat-sen Memorial Hospital, Sun Yat-sen University, Guangzhou, P. R. China; 5 Department of Pharmacology and Toxicology, School of Pharmaceutical Sciences, Sun Yat-sen University, HEMC, Guangzhou, P. R. China; Vanderbilt University Medical Center, United States of America

## Abstract

Endothelial injury related to oxidative stress is a key event in cardiovascular diseases, such as hypertension and atherosclerosis. The activation of the redox-sensitive Kv1.5 potassium channel mediates mitochondrial reactive oxygen species (ROS)-induced apoptosis in vascular smooth muscle cells and some cancer cells. Kv1.5 channel is therefore taken as a new potential therapeutic target for pulmonary hypertension and cancers. Although Kv1.5 is abundantly expressed in vascular endothelium, there is little knowledge of its role in endothelial injury related to oxidative stress. We found that DPO-1, a specific inhibitor of Kv1.5, attenuated H_2_O_2_-evoked endothelial cell apoptosis in an *in vivo* rat carotid arterial model. In human umbilical vein endothelial cells (HUVECs) and human pulmonary arterial endothelial cells (HPAECs), angiotensin II and oxLDL time- or concentration-dependently enhanced Kv1.5 protein expression in parallel with the production of intracellular ROS and endothelial cell injury. Moreover, siRNA-mediated knockdown of Kv1.5 attenuated, whereas adenovirus-mediated Kv1.5 cDNA overexpression enhanced oxLDL–induced cellular damage, NADPH oxidase and mitochondria-derived ROS production and restored the decrease in protein expression of mitochondria uncoupling protein 2 (UCP2). Collectively, these data suggest that Kv1.5 may play an important role in oxidative vascular endothelial injury.

## Introduction

Intact endothelium is important in the regulation of cardiovascular cell growth, cell migration, proliferation and apoptosis and vascular tone. In this regard, endothelial cell (EC) injury contributes to the impaired regulation of vascular tone, tissue perfusion, myocardial function, anticoagulant activity and inflammatory responses in vascular diseases and is thus a key characteristic pathophysiological feature of many cardiovascular diseases including atherosclerosis, hypertension, diabetic vasculopathy and heart failure. Large evidence has shown that chronic or acute reactive oxygen species (ROS) overproduction plays the critical role in endothelial insult and subsequent initiation of vascular remodeling [1]. Various cardiovascular stimuli, such as angiotensin II (Ang II) and oxidized low density lipoprotein (oxLDL) can trigger endothelial injury, which is accompanied by the increase in ROS production [1], [2], [3], [4]. On the other hand, exposure of vascular endothelium or ECs directly to ROS can induce cellular injury [5]. NADPH oxidase and mitochondria the two major sources of ROS generation, and both are highlighted in endothelial physiological cell signaling and pathophysiologic mechanisms [1]. Although ROS can modulate signaling pathways in endothelial cells at multiple levels, such as membrane receptors, channels, kinases and nuclear transcription factors, the precise mechanism underlying endothelial apoptosis related to oxidative stress is still not clear.

The Kv1.5 K^+^ channel, encoded by the *KCNA5* gene, is a member of the superfamily of voltage-gated potassium channels (Kv channels). Given its human atrial specificity and vital contribution to action potential formation [6], Kv1.5 is taken as a new pharmacological target for atrial fibrillation [7], [8], and thus many potent Kv1.5 inhibitors have been developed [9], such as (2-isopropyl-5-methylcyclohexyl) diphenylphosphine oxide (DPO-1) [10], mephetyl tetrazole (MT) [11], AVE0118 [9], AVE1231 [12]. In response to hypoxia, the activity of mitochondrial electron transport chain (ETC) is decreased and causes mitochondrial membrane potential (ΔΨ_m_) hyperpolarization, leading to reduction in mitochondrial hydrogen peroxide (H_2_O_2_) production [13]. The decrease in mitochondrial H_2_O_2_ generation directly suppresses Kv1.5 channel expression and activity, a critical process for the impairment of cellular apoptosis and hampers elimination of apoptotic cells, thus aggravating the progression of oxidative stress-related diseases such as PAH and cancer [14]. In contrast, overexpression of *KCNA5* gene or attenuation of Kv1.5 downregulation [15], [16], [17] reduces apoptosis during these diseases. Therefore Kv1.5 is taken as a potential therapeutic target for preventing the progression of PAH or cancers [14], [17], [18].

Although Kv1.5 is abundantly expressed in endothelial cells, little is known about its function in vascular endothelium. Given that Kv1.5 channel is highly redox sensitive and plays a key role in the cellular apoptosis in the various types of cells, we hypothesized that Kv1.5 was involved in endothelial cell injury related to oxidative stress. We found that DPO-1, a specific Kv1.5 inhibitor could significantly reduce apoptotic endothelial cells in an *in vivo* H_2_O_2_ -induced endothelial injury model. In addition, various vascular stimuli, like Ang II and oxLDL could concentration- or time-dependently induce Kv1.5 protein expression, intracellular ROS production and cell injury. In oxLDL-induced endothelial cell injury model, we further found that knockdown of *KCNA5* gene attenuated, whereas overexpression of KCNA5 gene enhanced oxLDL-induced endothelial morphological changes, ROS generation derived from NADPH oxidase and mitochondria, and the protein expression of uncoupling protein 2 (UCP2), a modulator of mitochondria-derived ROS production [19], [20]. Collectively, our results demonstrated that Kv1.5-ROS-mitochondria pathway is involved in endothelial injury related to oxidative stress.

## Materials and Methods

### Ethics Statement

The prior approval was obtained for human venous blood and human umbilical cord from the Medical Ethical Committee, the first Affiliated Hospital, Sun Yat-sen University. Adult blood samples were collected after written informed consent from healthy volunteers and umbilical cord after written informed consent from the mother after full-term pregnancies in accordance with the Declaration of Helsinki. Animal experiments were approved by Institutional Animal Care and Use Committee at Sun Yat-sen University (Approval No: IACUC-2009-0101) and were in accordance with the National Institutes of Health Guide for the Care and Use of Laboratory Animals.

### Reagents

Medium 200, Low serum growth supplement (LSGS) were purchased from Gibco, USA. All other reagents utilized were purchased from Sigma Chemical Co., USA unless otherwise specified.

### *In vivo* Detection of H_2_O_2_-induced EC Apoptosis in Rat Carotid Artery

Male Wistar rats aged 10–12 weeks (200±20 g, Experimental Animal Center, Sun Yat-sen University, China) were used in this study. The rats were randomly divided into three groups: a saline control group (Control), a H_2_O_2_-induced carotid arterial EC injury group (H_2_O_2_) and DPO-1 treatment group (DPO-1).The *in vivo* H_2_O_2_-induced EC apoptosis model was prepared as described previously [5]. Briefly, rats were anesthetized with pentobarbital (50 mg/kg, i.p.), then the right common carotid artery and the carotid bifurcation was exposed. The blood-stream was temporarily blocked by occlusion of the proximal part of common carotid arteries, distal internal and external carotid arteries with vascular clips. A PE10 catheter was placed into the common carotid artery via the external carotid artery. After blood was flushed with saline, the catheter was pulled back to just above the bifurcation and the lumen of the carotid artery was then flushed with saline containing 0.01 mM H_2_O_2_ for 5 min. After that, the external carotid artery was ligated, blood flow was restored by removing common and internal vascular clips, and the wound was closed at last. The DPO-1 group was given by intraperitoneal (i.p.) injection of DPO-1(0.3, 3 mg/kg) just after H_2_O_2_ flushing. At 6 h after the surgery, the rats were sacrificed, and the carotid artery was dissected and incised longitudinally and postfixed in 4% paraformaldehyde. The isolated arterial specimen was fluorescent stained with Hoechst 33342 (8 µg/ml in PBS). The specimen was viewed under confocal microscope and focused on the luminal endothelial side. The numbers of apoptotic cells and intact cells were counted in 4∼6 randomly chosen high-power fields (×400) for each specimen by an observer blinded to all the treatment.

### Cell Culture

Human pulmonary artery endothelial cells (HPAECs) were purchased from Cascade Biologics, Invitrogen (Carlsbad, CA, USA). Cells were propagated according to the instructions supplied. Briefly, cells were cultured in Medium 200 supplemented with LSGS. Passage 3∼6 HPAECs were used for experiments.

Human umbilical vein endothelial cells (HUVECs) were isolated and cultured as described previously [2]. Briefly, HUVECs were removed from human umbilical veins after collagenase type I digestion, and maintained at 37°C in 5% CO_2_ humidified atmosphere in medium 199 containing 20% fetal calf serum, penicillin (100 U/ml), streptomycin (100 U/ml), and heparin (50 U/ml), supplemented with L-glutamine (2 mM), sodium pyruvate (1 mM), and endothelial cell growth factor (β-ECGF, 5 ng/ml), on 0.1% gelatin–coated culture flasks. Endothelial cells were identified by their “cobblestone” mosaic appearance when reaching confluence and the presence of von Willebrand factor. Endothelial cell passages between 3 and 6 were used in the present studies.

### Isolation and Oxidation of LDL

Native LDL and oxLDL were prepared as previously described [2]. Briefly, Native LDL (d = 1.019–1.063) was isolated from fresh human plasma by sequential ultracentrifugation and oxidized by adding Copper sulphate (5 µM) for 24 hours. The thiobarbituric acid–reactive substance (TARS) content of oxLDL was detected (12.5±0.68 nM per 100 µg protein). The relative electrophoretic mobility of oxLDL vs. native-LDL was 2.1±0.3, arbitrary units. Lipoproteins were stored at 4°C in the dark and freshly prepared every 2 weeks.

### Observation of EC Injury through Inverted Microscopy Study and DAPI Staining

The cytomorphological changes of apoptotic cells were examined under inverted microscope (Nikon TS100, Japan). Images were captured randomly using Olympus digital camera (Olympus, Japan). For each sample, the representative image was chosen from at least five randomly chosen non-overlapping fields of view as previously described [2].

The EC injury in *in vitro* models was examined using DAPI staining as previously described [2]. ECs were fixed in 4% paraformaldehyde for 15 min, and then incubated with 2.5 µg/ml of DAPI (Molecular Probes, USA) for 30 min at room temperature. The pictures of photographed nuclei were then acquired in laser scanning confocal microscope (FV500, Olympus, Japan). Cells with condensed chromatin or shrunken, irregular, or fragmented nuclei were considered apoptotic. Apoptotic values were calculated as the percentage of apoptotic cells relative to the total number of cells in each randomly chosen non-overlapping fields of view (>200 cells).

### Western Blotting

Cells were scraped from 6-cm petri dishes and centrifuged to collect the pellets. Cells pellets were lysed in 100 µl of ice-cold lysis buffer consisting of 125 mm Tris-HCl (pH 7.4), 10% (v/v) glycerol, 2% (w/v) SDS, 1×protease inhibitor cocktail (Merck, USA) and 100 µg/ml PMSF. The cell lysates were then centrifuged at 12,000 rpm for 10 min, and the supernatant was collected. The protein concentrations in the supernatant were assayed by the BCA kit (Beyotime, China). The protein samples (40 µg) were separated on an 8% SDS-PAGE gel and transferred onto PVDF membranes by using Mini Trans-Blot cell transfer apparatus (Bio-Rad, USA). The membranes were probed with antibodies against Kv1.5 (Alomone Labs, USA) or UCP2 (Biolegend, USA), respectively. The targeted proteins were detected using an enhanced chemiluminescence detection system (Beyotime, China).

### RNA Interference

Small interfering RNA (siRNA) oligonucleotide sequences were designed and purchased from Qiagen, USA. The sense strand of the siRNA used to silence the *KCNA5* gene was 5′-CGGACGAGAUACGCUUCUATT-3′, corresponding to Target Sequence 5′-CGCGGACGAGATACGCTTCTA-3′ (Gene ID: 3741). The AllStars Negative Control (Qiagen, USA) was used as control siRNA. The siRNAs were transfected into HPEACs by using HiPerFect Transfection kit (Qiagen, USA) according to the manufacturer's protocol. HPEACs were seeded in 6-well plates (6×10^4^ cells/well) and grown for 24 h. 37.5, 75, 150 ng of siRNA was respectively diluted in 100 µl Medium 200 without serum (this will give a final siRNA concentration at 2.5, 5, 10 nM) and mixed by vortexing. Then 12 µl of HiPerFect Transfection Reagent was added to the diluted siRNA. All the complexes were incubated for 5 min at room temperature, and added onto the cells. After 3 h, 1600 µl of culture medium was added and incubated for further 48 h.

### Adenoviral Infection Experiments

The plasmid hKv1.5-pCMS-EGFP (generously gifted from Jason X.-J. Yuan, Department of Medicine, University of California, San Diego) was used to construct recombinant flag-tagged adenoviral vector to overexpress human *KCNA5* (Adv-Kv1.5) by Shanghai GeneChem Co., Ltd, China. The recombinant viruses were stored at –80°C. The adenoviral infection experiments were carried out according to the manufacturer's instruction. The flag-tagged adenoviral vector (Ad-Con) without expressing *KCNA5* was employed as the negative control.

### Measurement of Intracellular ROS

Intracellular ROS was detected as described previously [2]. Briefly, intracellular ROS was measured by laser scanning confocal microscopy using 2′,7′-dichlorofluorescin diacetate (DCFH-DA, SIGMA, USA) as a ROS-sensitive fluorescence probe. When DCFH is formed inside cells, it will be oxidized by intracellular ROS and converted to DCF, and therefore the detected fluorescent signal will be proportional to ROS production. The DCFH-DA was added 20 min prior to Ang II or ox-LDL treatment.

### Measurement of Mitochondrial ROS Generation

Intracellular measurement of mitochondrial ROS generation was performed using MitoSOX Red (Molecular Probes, USA) as previously described [21]. MitoSOX Red is a fluorescent dye specific for the detection the mitochondrial superoxide in live cells. Briefly, the cells were washed with Hank’s balanced salt solution (HBSS) and loaded with MitoSOX (2 µM) for 10 min. To confirm the mitochondrial localization of MitoSOX Red, HPEACs were also incubated with MitoTracker Green (0.2 µM, Invitro-gen, USA) for 10 min. Cells planted in 24-plates were then treated with oxLDL for 1 h following the removal excess fluorescent dyes with HBSS, and imaged in a laser confocal scanning microscopy (FV500, Olympus, Japan, excitation/emission: 510/580 nm). The fluorescence intensity from cells planted in 96-plates were Quantified by a SpectraMax GEMINI EM fluorescent plate reader (Molecular Devices, USA, excitation/emission: 485/538 nm). The mitochondrial ROS levels per well were expressed as the ratio of MitoSOX/Mitotracker to compensate for differences of mitochondrial mass and unequal MitoSOX loading, and the average ratio was calculated from over six coordinate wells of each group.

### Statistics

Data are presented as means ± S.E.M. Statistical comparisons were made using one way ANOVA followed by a posthoc comparison using the least significant difference test (SPSS 11.0). Values of *P*<0.05 were considered statistically significant.

## Results

### Effects of Kv1.5 Inhibitor, DPO-1 on H_2_O_2_-induced EC Apoptosis in Rat Carotid Arteries

**Figure 1:**
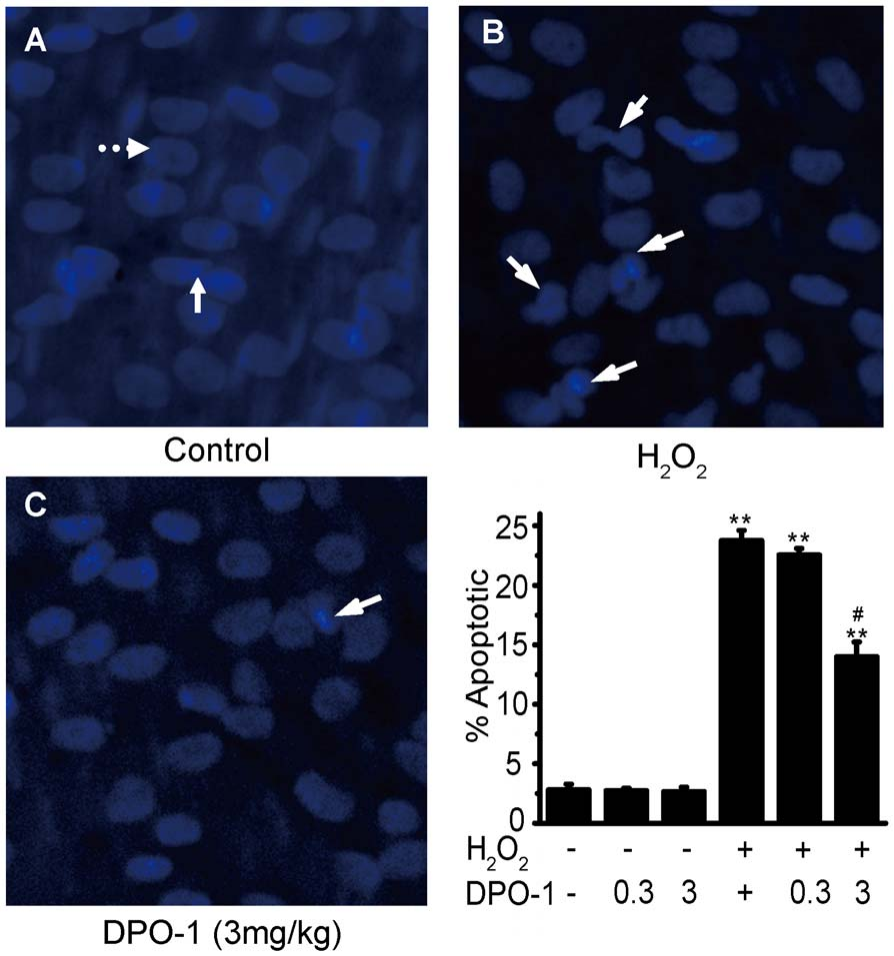
Kv1.5 inhibitor, DPO-1 attenuated H_2_O_2_-induced *in vivo* EC apoptosis in rat carotid artery. DPO-1(0.3, 3 mg/kg) was i.p. dose administrated at 6 hours after H_2_O_2_ injury. Representative morphological changes of apoptotic ECs were determined by en face Hoechst 33342 staining in Control (A), H_2_O_2_ injury (B) and DPO-1 treatment (C) groups. The contralateral side of carotid artery at 6 h after injury served as control, nuclear morphology of normal ECs (round, shown by full line arrow in (A)) and VSMCs (spindle-shaped, shown by dotted line arrow in (A)) can be observed. In (B) & (C), the white arrows show typical apoptotic morphological changes in apoptotic ECs, including chromatin condensation and nuclear fragmentation. The values were presented as ± SEM of 6 independent experiments. * *P*<0.01 vs. control, # *P*<0.01 vs. H_2_O_2_ group.

Kv1.5 channel is ubiquitously expressed, and in vascular smooth muscle cells it has been related with regulation of vasoconstriction, cell proliferation and apoptosis [14]. To exclude the influence of Kv1.5 from vascular smooth muscle cells, a rat model in which hydrogen peroxide (H_2_O_2_) induces EC apoptosis in luminal endothelial side of carotid artery [5] was adapted to examine whether Kv1.5 is involved in EC injury *in vivo*. In agreement with the previous study [5], 0.01 mM H_2_O_2_ significantly caused EC damage, but had no effects on vascular smooth muscle cell, as illustrated by the characteristic morphological appearance of apoptotic ECs: chromatin condensation, nuclear fragmentation, and apoptotic bodies (full line arrow in Fig. 1). DPO-1 is a selective inhibitor of Kv1.5 channels, which exhibits inhibitory effects on ultrarapid delayed rectifier current (*I*_Kur_) encoded by Kv1.5 within the dose range of 1∼12 mg/kg (i.v) in cardiac myocytes in rat, canine and African green monkey models [22]. We therefore examined the effect of DPO-1 (0.3, 3 mg/kg, i.p.) on the *in vivo* EC apoptosis model, and found that DPO-1 at a dose of 3 mg/kg significantly reduced H_2_O_2_-induced EC apoptosis from 24±1% to 14±1% (*P*<0.01, n = 6, Fig. 1C).

### Effects of Ang II and oxLDL on Kv1.5 Protein Expression, Intracellular ROS Production, and EC Injury in Vascular Endothelial Cells

Ang II and oxLDL are important vascular factors that mediate endothelial injury and endothelial dysfunction during atherogenesis, inflammation and vascular remodeling in many cardiovascular diseases. Intracellular ROS overproduction has been recognized to play a key role in these processes [1], [3], [4], [23], [24].

**Figure 2:**
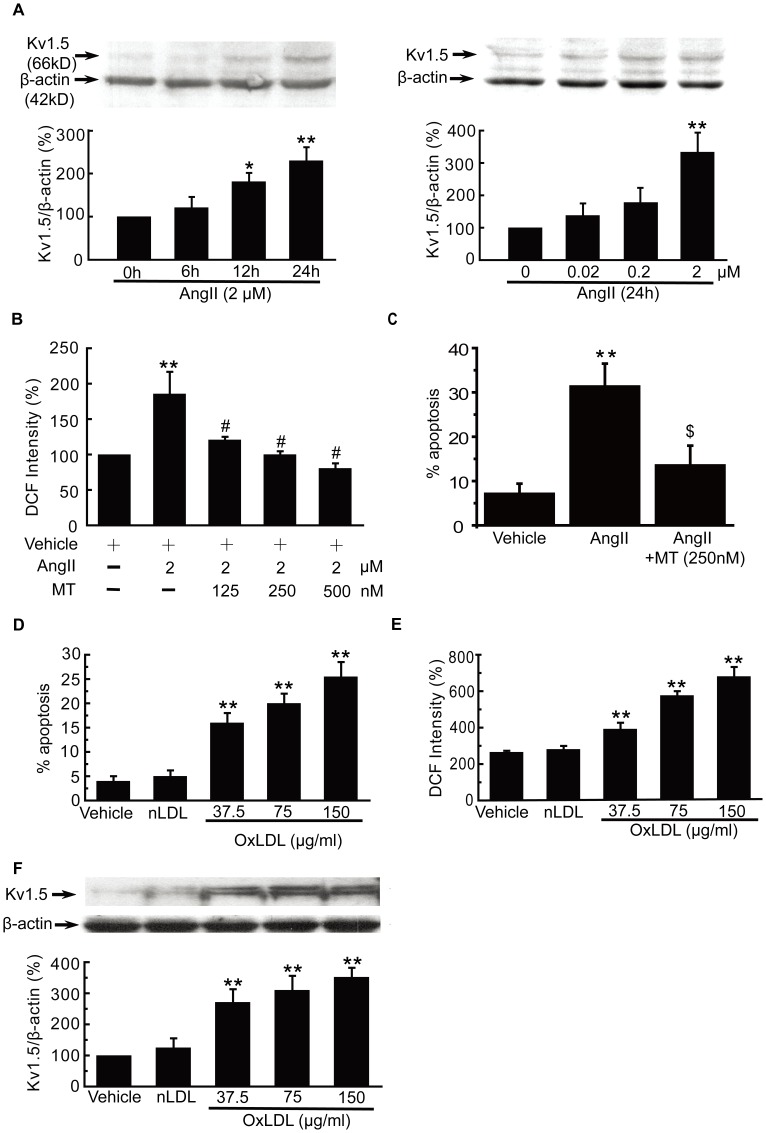
Ang II and oxLDL affected Kv1.5 protein expression, intracellular ROS production, and endothelial cell injury. HUVECs were incubated with AngII at different concentrations for different times. Ang II time (A)- or concentration (B)-dependently enhanced the Kv1.5 protein expression. Pretreatment with MT for 30 min inhibited Ang II (2 µM, 24 h)-induced increase in intracellular ROS levels in HUVECs in a concentration-dependent manner (C), as determined by DCF fluorescence. MT also significantly attenuated Ang II (2 µM, 24 h)-induced HUVEC injury. Incubation of HPAECs with oxLDL at the concentration of 37.5, 75 and 150 µg/ml significantly increased EC injury (D), intracellular ROS production (E) and Kv1.5 protein expression (F) in a concentration-dependent manner. The values were presented as ± SEM of 6 independent experiments for Ang II- or oxLDL-treatment, respectively. * *P*<0.05, ** *P*<0.01 vs. control; $ *P*<0.05, # *P*<0.01 vs. Ang II group.

To determine whether Ang II has effects on Kv1.5 protein expression, we incubated HUVECs with Ang II at different concentrations for different times. Ang II time- or concentration-dependently increased the Kv1.5 protein expression. Incubation HUVECs with 2 µM Ang II for 12 h and 24 h significantly enhanced the Kv1.5 protein expression (178±45% and 334±60% of control HUVECs, respectively, Fig. 2A), and incubation with Ang II (0.2 µM and 2 µM) for 12 h significantly enhanced the Kv1.5 protein expression (181±20% and 230±31% of control HUVECs, respectively, Fig. 2A). Growing evidence has shown that pleiotropic vascular effects of Ang II were mediated through intracellular ROS generation [4], [23], [24]. As shown in Fig. 2B, Ang II (2 µM, 24 h) caused a significant increase in intracellular ROS production (183.7±10.5 vs. control group), as determined by DCF. The increase was inhibited by the pretreatment with another selective Kv1.5 channel inhibitor MT for 10 min, in a concentration-dependent manner. MT at 125, 250, 500 µM significantly inhibited Ang II- induced intracellular ROS production to 120.8±4.2%,100.1±4.5%,80.6±6.9% vs. control group,respectively, indicating that the effect of Ang II on intracellular ROS production may be mediated through Kv1.5. Within the dose range of 0.1 to 10 µM, Ang II can induce endothelial cell apoptosis [25]. Consistent with literatures, Ang II (2 µM, 24 h) induced EC injury (Fig. 2C), in parallel with the increase in Kv1.5 protein expression and overproduction of intracellular ROS levels. Compared with control, 2 µM Ang II could increase the percentage of apoptotic cells up to 200.2±16.7% vs. control, and preincubation with MT (250 nM) markedly reduced the percentage of Ang II-induced apoptotic HUVECs to 124.1±36.0% (*P*<0.05, Fig. 2C).

The oxLDL-induced EC injury model has been applied to mimic the oxidative endothelial injury during atherogenesis, which has well been established in our previous study [2]. In agreement with our previous report, incubation of HPAECs with oxLDL at the concentration of 37.5, 75 and 150 µg/ml significantly increased EC injury, ROS production in a concentration-dependent manner (Fig. 2D&E). Moreover, oxLDL at these concentrations simultaneously enhanced Kv1.5 protein expression in a concentration-dependent manner (270±40, 310±45 and 350±30% of control HPAECs, respectively, *P*<0.05, Fig. 2F), whereas native LDL (nLDL, 150 µg/ml) did not exhibit any effects on EC injury, ROS production and Kv1.5 protein expression (*P*>0.05, Fig. 2D,E,F ).

### Effects of Kv1.5 siRNA and Adenoviral Kv1.5 Gene Transfer on Kv1.5 Protein Expression

**Figure 3:**
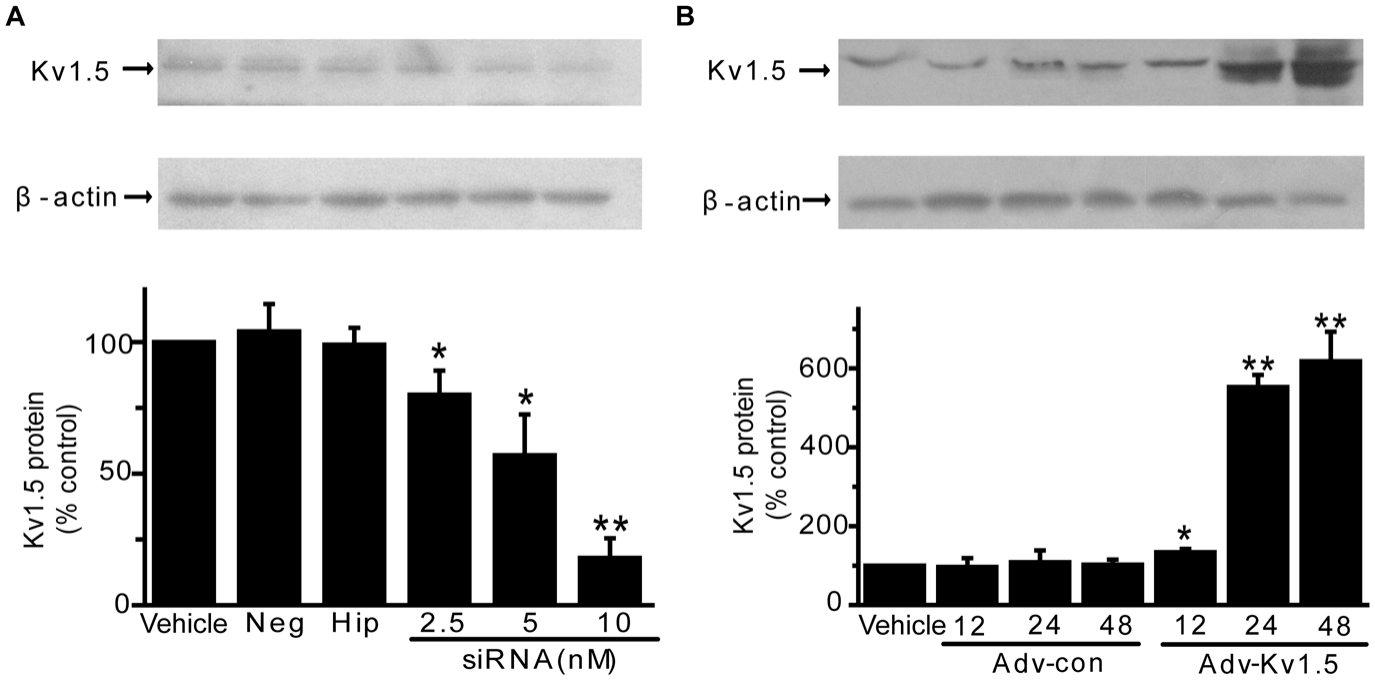
Effects of Kv1.5 siRNA adenoviral Kv1.5 overexpression on endogenous Kv1.5 protein expression in HPAECs. (**A**). HPAECs were treated with 2.5, 5, 10 nM Kv1.5 siRNA for 48 h and Kv1.5 protein expression was analyzed by western blot. * *P*<0.05, ** *P*<0.01 vs. Vehicle control, n = 3. (**B**). HPAECs were infected with adnovirus containing *KCNA5* (Ad-Kv1.5) and control–adnovirus (Ad-con) for 12, 24, 48 h. * *P*<0.05, ** *P*<0.01 vs. Ad-con at corresponding time points, n = 3.

Although the above applied DPO-1 or MT were previously described as potent and selective Kv1.5 inhibitors, they also inhibit other Kv channels but to a less extent, such as I_K1_ and I_Ks_ [26]. To identify the function of Kv1.5 in vascular EC injury related to oxidative stress, we constructed adenovirus containing human Kv1.5 and siRNA specific for human Kv1.5. Kv1.5 siRNA (2.5, 5, 10 nM) effectively reduce the endogenous level of Kv1.5 protein expression to 80±8%, 57±15% and 18±7% of the negative control group (Fig. 3A), the transfection of 10 nM siRNA in ECs for 48 h was thus fixed in the following siRNA interference experiments. Compared with adenoviral mock vector group (Ad-con), there was 0.4-, 4.5-, 6.2-fold increase in Kv1.5 protein expression at doses of 10^4^ PFU/ml for 12, 24, 48 h, respectively (Fig. 3B). Accordingly, adenoviral Kv1.5 gene transfer at the dose of 10^4^ PFU/ml was used in subsequent experiments.

### Kv1.5 Modulates oxLDL-induced EC Injury and Intracellular ROS Generation

**Figure 4:**
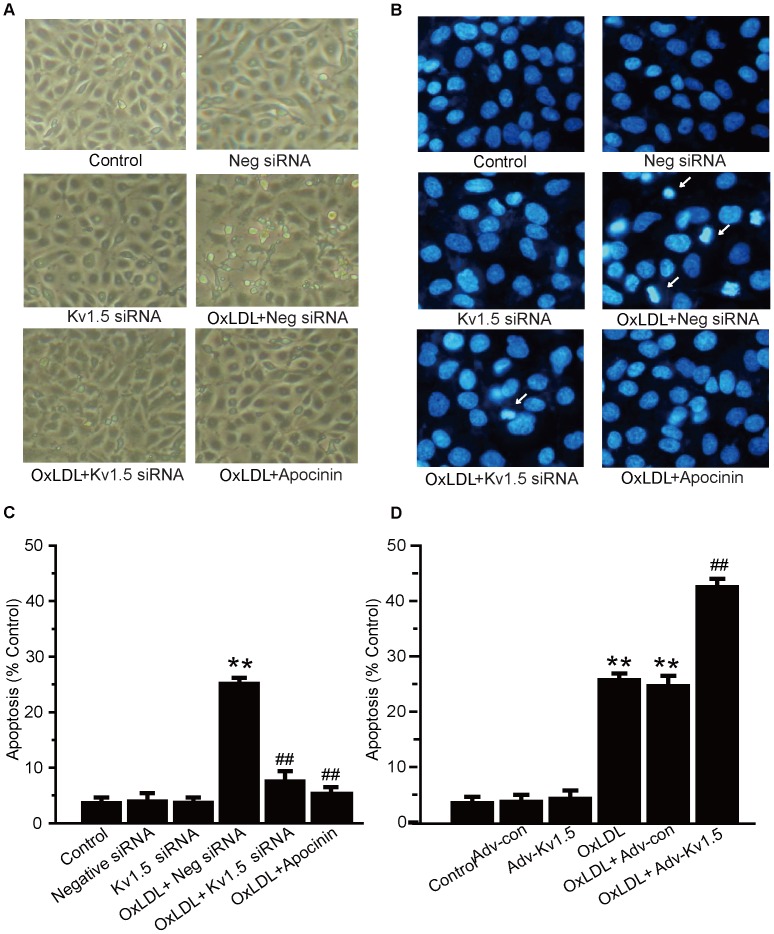
OxLDL-induced EC injury was attenuated by Kv1.5 siRNA, but aggravated by adnoviral Kv1.5 overexpression. HPAECs were transfected with Kv1.5 siRNA (10 nM) or control siRNA for 48 h, or infected with adnovirus containing *KCNA5* and control–adnovirus for 24 h, then treated with oxLDL (150 µg/ml) for further 24 h. The oxLDL-induced EC injury was detected by optical microscopy and DAPI staining. The optical microscope observation (A) and DAPI staining (B) show the morphological changes in Kv1.5 siRNA pretreated HPAECs. The mean values of percentages of apoptotic cells were summarized after DAPI staining in Kv1.5 siRNA (C)- and *KCNA5* adnovirus- pretreated (D) HPAECs. The values are presented as means ± SEM of 6 independent experiments. * *P*<0.05, ** *P*<0.01 vs. Vehicle control; # *P*<0.05, ## *P*<0.01 vs. Ad-con.

Confluent HPEACs had a “cobblestone” mosaic appearance (Fig. 4A). Incubation with oxLDL (150 µg/ml) for 24 h induced typical morphological change of injured ECs as evidenced by cell retraction and rounding up, formation of cellular fragments. DAPI staining was also employed to study late stage morphological indicators of cellular apoptosis, such as endothelial cell shrinkage, nuclear segmentation and chromatin condensation. The Kv1.5 siRNA significantly attenuated oxLDL-induced morphological changes (Fig. 4A). DAPI staining data additionally showed that the Kv1.5 siRNA significantly reduced the percentage of oxLDL-induced apoptotic HPEACs by about 70% (Fig. 4B&C). In contrast, the Ad-Kv1.5 infection exaggerated oxLDL-induced apoptosis. (Fig. 4D).

**Figure 5:**
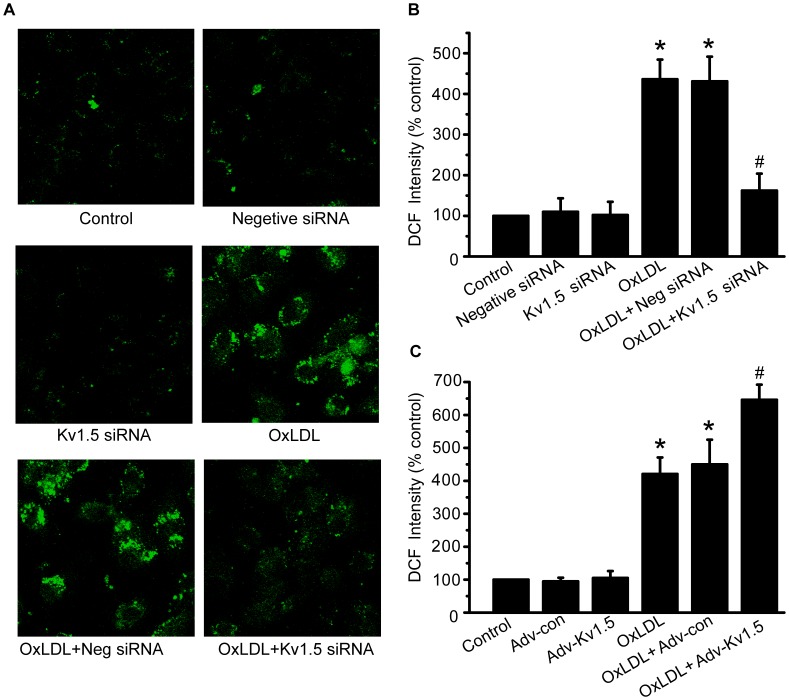
OxLDL-induced ROS overproduction was attenuated by Kv1.5 siRNA, but exaggerated by Kv1.5 overexpression. Intracellular ROS levels were detected using DCFH-DA staining in HPAECs transfected with Kv1.5 siRNA (10 nM) or infected with adnovirus containing *KCNA5*, which were treated with oxLDL (150 µg/ml) for further 1 h. (**A**) & (**B**). Kv1.5 siRNA significantly attenuated oxLDL-induced endothelial ROS overproduction in HPAECs, as demonstrated by representative images from laser scanning confocal microscopy and bar graph showing fluorescence intensity measured in Multi-Mode Microplate Reader. (**C**). Bar graph shows that adnoviral *KCNA5* gene transfer exaggerated oxLDL-induced endothelial ROS overproduction in HPAECs. The values are presented as means ± SEM of 6 independent experiments. * *P*<0.05, ** *P*<0.01 vs. Vehicle control; # *P*<0.05, ## *P*<0.01 vs. Ad-con.

The excessive ROS generation was shown in oxLDL-induced EC injury in the present study and many previous studies [2]. As shown in Fig. 5A&B, knockdown of Kv1.5 with siRNA transfection significantly attenuated oxLDL-induced increase in ROS levels (162.3±41.8% vs. 436.1±48.4% of control group, *P*<0.05). On the contrary, Kv1.5 protein overexpression with adv-Kv1.5 infection further enhanced oxLDL-induced increase in ROS generation by about 23% (646.0±46.1% of oxLDL group, *P*<0.05, Fig. 5C). These data provide the direct evidence that Kv1.5 is important for regulating oxLDL-induced EC apoptosis through elevation of intracellular ROS levels.

### Kv1.5 Regulates oxLDL-induced Endothelial Mitochondrial ROS Generation and UCP2 Protein Expression

**Figure 6:**
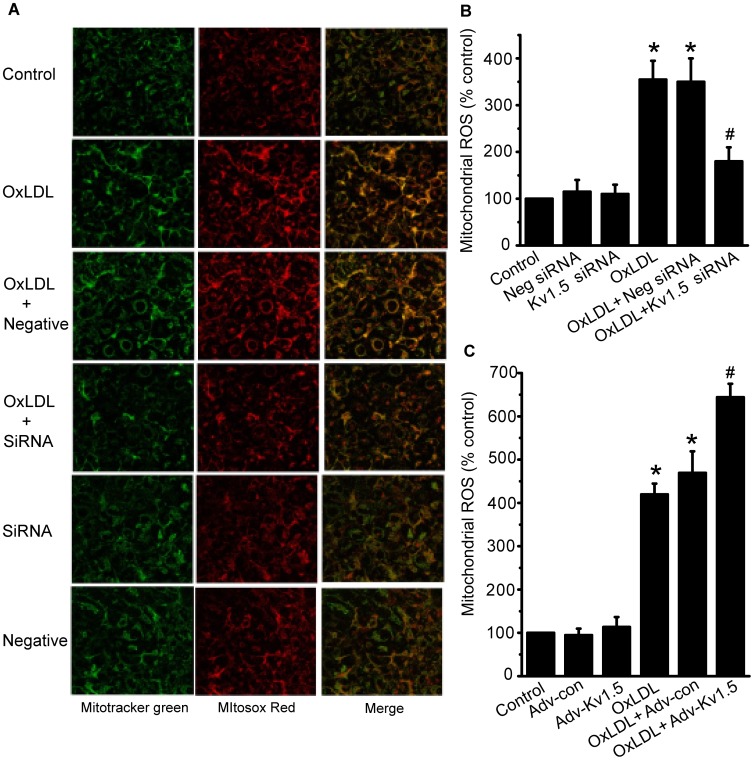
OxLDL-induced mitochondrial ROS overproduction was attenuated by Kv1.5 siRNA, but exaggerated by Kv1.5 overexpression. The mitochondrial ROS generation was detected using MitoSOX reagents in HPAECs transfected with Kv1.5 siRNA (10 nM) or infected with adnovirus containing *KCNA5*, which were treated with oxLDL (150 µg/ml) for further 1 h. (**A**) The representative images from laser scanning confocal microscopy Kv1.5 siRNA significantly attenuated oxLDL-induced mitochondrial superoxide production in HPAECs. (**B**) & (**C**). The mitochondrial ROS levels in HPAECs transfected with Kv1.5 siRNA (B) or infected with adnovirus overexpression of Kv1.5 (C) were quantified by the fluorescent plate reader and expressed as the ratio of MitoSOX to Mitotracker. The values are presented as means ± SEM of 6 independent experiments. * *P*<0.05, ** *P*<0.01 vs. Vehicle control; # *P*<0.05, ## *P*<0.01 vs. Ad-con.

Previous studies in pulmonary arterial smooth muscle cells and some cancers have shown that mitochondria-derived ROS (mainly H_2_O_2_) is critical for activation or expression of Kv1.5 channels [14]. The production of mitochondrial ROS is also important for oxLDL-induced EC apoptosis, which has been implicated in atherogenesis in cardiovascular diseases [27], [28]. Consistent with previous report [27], incubation HPAECs with oxLDL (150 µg/ml) for 1 h significantly increased the mitochondrial ROS level. Remarkably, this increased mitochondrial ROS production was diminished to levels comparable to that of control, following treatment with Kv1.5 siRNA (Fig. 6A&B). In contrast, Kv1.5 overexpression further enhanced the oxLDL-induced mitochondrial ROS production to a significant extent (Fig. 6C), the null adenoviral vector and negative siRNA had no effects on oxLDL-induced mitochondrial ROS production (*P*>0.05). These data demonstrated that Kv1.5 can regulate oxLDL-induced HPAEC apoptosis via mitochondrial ROS production.

**Figure 7:**
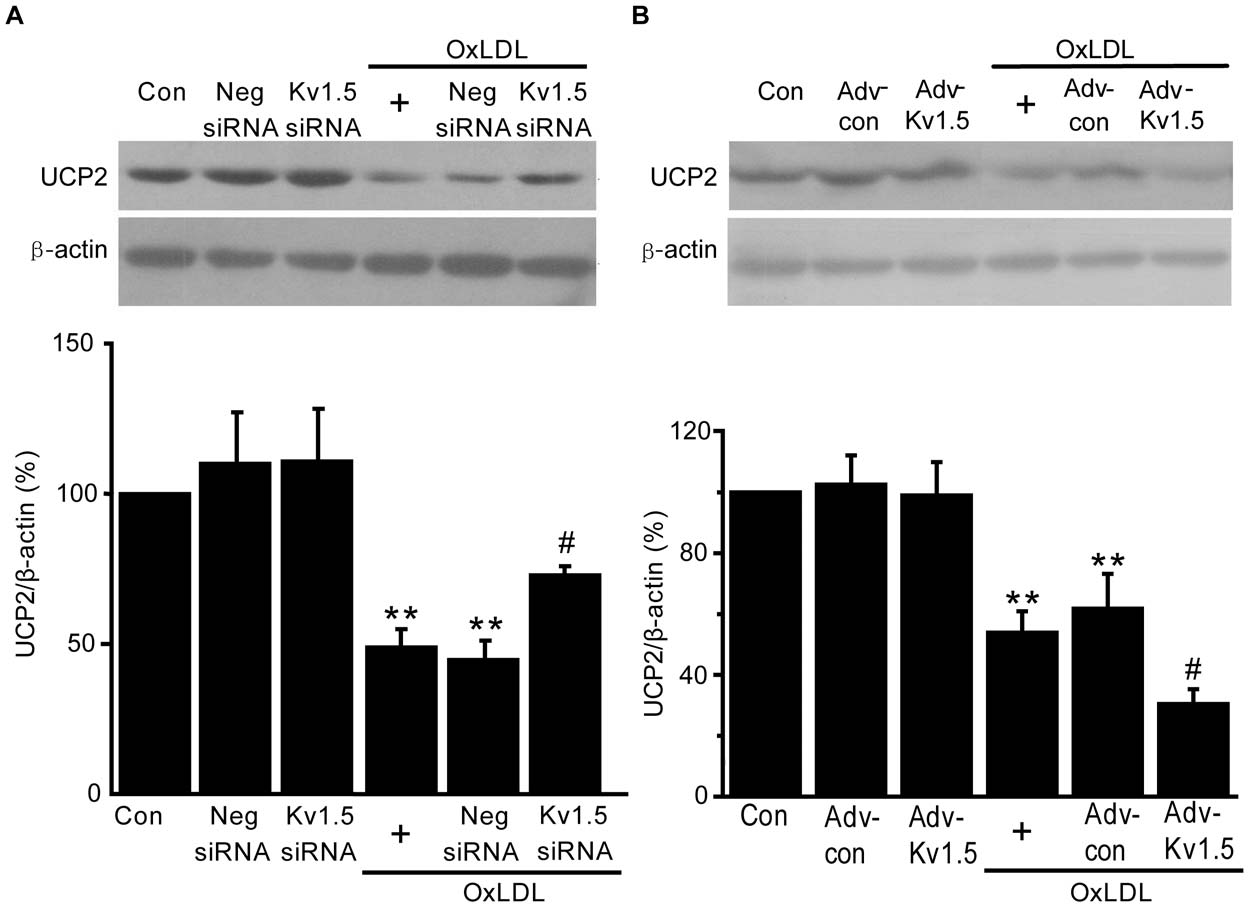
OxLDL-induced reduction in UCP2 protein was restored by Kv1.5 siRNA, but exaggerated by Kv1.5 overexpression. The UCP2 protein expression was detected by western blot in HPAECs transfected with Kv1.5-siRNA (A) and or infected with adnovirus containing *KCNA5* (B). Digital photographs showing UCP2 protein expression are on the top panel and bar graph showing quantitative analysis of the total protein are on the lower panel. The values are presented as means ± SEM of 6 independent experiments. ** *P*<0.01 vs. corresponding control group; # *P*<0.01 vs. oxLDL group.

UCP2 is an important mitochondrial membrane protein that regulates mitochondrial membrane potential (ΔΨ_m_) and thus modulates mitochondrial ROS production [19]. OxLDL significantly decreased UCP2 protein expression in HPAECs (44.8±6.3% of the control group, *P*<0.01, Fig. 7A) Kv1.5 knockdown by siRNA significantly increased endothelial UCP2 protein expression (72.9±3.2% of control group, *P*<0.01, Fig. 7A). In contrast, Kv1.5 overexpression further decreased endothelial UCP2 expression (29.5±4.9% of control group, *P*<0.01, Fig. 7B). These data demonstrate that UCP2 is important for oxLDL-induced endothelial injury.

## Discussion

In this study, we have investigated the role of Kv1.5 in vascular endothelial cell injury related to oxidative stress. We found that DPO-1, a specific Kv1.5 inhibitor, significantly attenuated H_2_O_2_-evoked endothelial cell apoptosis in an *in vivo* rat carotid arterial model. Furthermore, Kv1.5 was abundantly expressed in HUVECs and HPAECs. The well-known vascular stimuli, Ang II and oxLDL, could concentration- or time-dependently induce endothelial cell injury, accompanied by an increase in Kv1.5 protein expression and intracellular ROS production. To further confirm the importance of Kv1.5 for EC injury process, we applied adenovirus–mediated Kv1.5 gene transfer and siRNA specific for human Kv1.5 aiming to regulate endogenous Kv1.5 protein in EC models. In oxLDL-induced endothelial cell injury model, transfection of Kv1.5 siRNA into HPAECs could attenuate cellular injury, increase in intracellular ROS levels as well as mitochondrial ROS levels and restore the downregulation of UCP2 protein. In contrast, adenoviral Kv1.5 gene transfer exhibited the opposite effects. These results provide the compelling evidence that Kv1.5 plays an important role in endothelial cell injury related to oxidative stress.

The increase in oxidative stress by different vascular risk factors is a key mechanism of endothelial injury related to chronic endothelial dysfunction., Among the reactive oxygen species, superoxide and H_2_O_2_ are the major ones to mediate oxidative stress-induced endothelial damage [1]. Superoxide is always the first ROS generated in the oxygen free chain during the early phase of endothelial cell injury induced by different vascular risk factors. Superoxide is unstable and rapidly dismutated to H_2_O_2_ [1]. H_2_O_2_ is more stable than other ROS and easily to penetrate plasma membrane, and therefore commonly used to create oxidative stress-induced damage models [1], [5]. In an *in vivo* H_2_O_2_ -induced endothelium injury model [5], we found that DPO-1 (3 mg/kg, i.p.) significantly improved endothelial cell apoptosis. This dosage is within the dose range that DPO-1 exhibited significant selective inhibition on *I*_Kur_ in cardiac myocytes in various *in vivo* models including the rat model [22], [29]. Moreover, this *in vivo* H_2_O_2_ -induced endothelium injury model is well established to observe the effects of Kv1.5 inhibitor on endothelial cell apoptosis exclusively [5], [30], and the influence of Kv1.5 in vascular smooth muscle cells would be avoided. Accumulating evidence have shown that several angiotensin type I receptor blockers can inhibit Kv1.5 channel activity and ultrarapid delayed rectifier K^+^ current (*I*_Kur_) in cardiac myocytes [31], [32]. We here investigated whether Ang II can directly induce Kv1.5 protein expression. In the Ang II (2 µM)-induced endothelial cell apoptosis model [25], Ang II induced Kv1.5 protein expression, in parallel with endothelial cell injury and increase in intracellular ROS generation. Pretreatment of endothelial cells with MT, another Kv1.5 channel inhibitor prevented all these effects. In another endothelial cell injury model induced by oxLDL, which mimics endothelial injury in atherogenesis [2], [33], [34], we got the similar findings that oxLDL could induce Kv1.5 protein expression, ROS overproduction and endothelial apoptosis within the same concentration range. Thus, Kv1.5 has an important role in endothelial cell injury by different cardiovascular stimuli because siRNA-mediated knockdown of Kv1.5 attenuated oxLDL-induced endothelial cell injury while adenovirus-mediated Kv1.5 overexpression exaggerated that.

NADPH oxidase is the major source of ROS generation in physiology and pathophysiology of endothelial cells. Increased NADPH oxidase activity and genetic disruption of components in NADPH oxidase complex has been detected in atherosclerosis, hypertension and many other diseases [35]. Due to its indiscriminate nature to various free radicals, DCF-DA is widely used to quantify overall intracellular ROS [2]. Many groups, including ours [2], have shown that oxLDL induces rapid generation of ROS through activation of NADPH oxidase. Interestingly, both apocynin, an inhibitor of NADPH oxidase and Kv1.5 siRNA dramatically attenuated endothelial cell injury and intracellular ROS generation in response to oxLDL, whereas, adenoviral Kv1.5 overexpression enhanced oxLDL-induced oxidative injury. Together with our previous findings, we believe that regulation of Kv1.5 in oxLDL-induced endothelial cell injury requires the NADPH oxidase.

The mitochondrial dysfunction and ROS overproduction plays a critical role in vascular endothelial injury [4], [36], [37], [38]. Increased levels of oxidatively modified lipoproteins in blood circulation have been shown to induce mitochondrial dysfunction and subsequent endothelial apoptosis [39]. Exposure of vascular endothelial cells to oxLDL significantly reduced mitochondrial complex specific oxygen consumption and key enzyme activities in respiratory chain complexes I–IV, led to the significant increase in mitochondrial-associated ROS [37]. Consistent with these literatures, we found that oxLDL significantly enhanced the mitochondrial ROS generation. The oxLDL-mediated increase in mitochondrial ROS could be prevented by siRNA specific to Kv1.5 and further enhanced by adnovirus-mediated Kv1.5 overexpression. These data indicate that Kv1.5 also regulates cell apoptosis signaling through mitochondria-dependent ROS generation which is in agreement with previous findings in PAH and cancers. Indeed, Chronic hypoxia-induced downregulation of Kv1.5 is a common feature in pulmonary vascular smooth muscle cells and cancer cells. In addition, hyperpolarization of ΔΨ_m_ can suppress the release ofH_2_O_2_ from mitochondria and downregulate Kv1.5, impeding subsequent apoptosis cascade in experimental PAH and cancer cells. The mitochondrial abnormality can be reversed by dichloroacetate or Kv1.5 overexpression [14], [17]. Although Kv1.5 is indicated as the downstream target of mitochondrial ROS generation in cell apoptosis in PAH and cancer cells [14], [17], the present study could not answer whether it is the case in endothelial cell injury.

UCP2 is a member of the mitochondrial anion carrier family and located in the mitochondrial membrane. Large evidence support that UCP2 is a key protein to modulate ΔΨ_m_ and subsequent mitochondria-derived ROS production [40], [41]. Recent study indicated that UCP2 overexpression reduced intracellular ROS production in the en face endothelium of aorta and mesenteric artery of diet-induced obese mice, whereas UCP2 deficiency enhanced ROS production [42]. In human aortic endothelial cells, it has been demonstrated that UCP2 decreased ROS generation by reducing ΔΨ_m_ and increasing the rate of electron transport, and thus inhibited endothelial apoptosis induced by lysophophatidylcholine [43]. UCP2 deficient mice displayed significant endothelial dysfunction, increase in ROS production and severe atherosclerotic lesions [36]. We here investigated whether UCP2 is involved in oxLDL-induced endothelial cell injury and is regulated by Kv1.5. We found that oxLDL significantly reduced UCP2 protein expression. Interestingly, knockdown of Kv1.5, could partially restore whereas Kv1.5 overexpression could enhance oxLDL-induced downreglution of UCP2 protein expression. The Kv1.5-mediated modulation of UCP2 expression is in parallel to its regulation on ROS derived from mitochondria and intracellular spaces. Although UCP2 is indicated as an important mitochondrial membrane protein, additional studies on how modulation of Kv1.5 can affect UCP2 expression and in turn regulate intracellular oxidative stress and cell apoptosis are necessary to understand the mechanism of endothelial cell injury and dysfunction. In breast tumor cell lines, loss of TGF-β/SMAD dependent signaling could promote UCP2 expression; facilitate reduction of mitochondrial ROS production and thus augmenting tumor cell survival and proliferation [44], [45]. Indeed, the SMAD4 expression is often lower in breast cancer than in the surrounding nonmalignant epithelium [46], suggesting that SMAD4 may play a pivotal role in mitochondrial ROS-related cell survival [45]. SMAD4 belongs to the highly conserved Darfwin family of proteins, which bind to receptor-regulated SMADs such as SMAD1 and SMAD2, and form transcriptional complexes to modulate TGF-β gene. Ang II can enhance the expression of TGF-β1, P-SMAD2/3 and SMAD4, which may serve as a key mechanism underlying atrial fibrillation (AF)-induced atrial fibrosis [47]. Kv1.5 is well known as a drug target against AF. OxLDL has also been found to enhance TGF-β/SMAD signaling in the oxidative progression of glomerular diseases [48]. Hence, it is possible that Ang II- or oxLDL-induced increase in Kv1.5 may interact with TGF-β/SMAD signaling pathway, especial with SMAD4, which can reduce UCP2 expression, enhance mitochondrial ROS production and promote endothelial injury. Although this hypothesis is in good accordance with the findings in cancer cells, much more research will be needed. Nevertheless, the restoration of Kv1.5 siRNA on oxLDL-induced reduction of UCP2 protein expression implies that UCP2 may be an important mitochondrial target in endothelial Kv1.5-ROS signaling cascade.

In summary, we here have demonstrated that various vascular stimuli induced Kv1.5 protein expression in endothelial cell injury through increasing oxidative stress levels; and knockdown of Kv1.5 attenuated, whereas Kv1.5 protein overexpression enhanced oxLDL-induced endothelial morphological changes, ROS generation derived from NADPH oxidase and mitochondria, and UCP2 protein expression. The present study for the first time suggested that Kv1.5 play an important role in oxidative stress-related vascular endothelial cell injury. The Kv1.5 regulating mechanism may involve ROS generation derived from intracellular space and mitochondria, as well as UCP2 protein expression. Given that endothelial injury is a key event in endothelial dysfunction in many pathophysiological processes, these new finding provides a new mechanism underlying oxidative endothelial cell injury.
